# Successful Termination of Insulin Therapy in Transient Neonatal Diabetes Mellitus

**DOI:** 10.1155/2023/6667330

**Published:** 2023-12-11

**Authors:** Risa Sakai, Nobuyuki Kikuchi, Daisuke Nishi, Haruko Horiguchi

**Affiliations:** Department of Pediatrics, Yokohama Rosai Hospital, 3211 Kozukuecho, Kohoku-ku, Yokohama, Kanagawa 222-0036, Japan

## Abstract

A sensor-augmented pump (SAP) therapy is used to treat neonatal diabetes mellitus (NDM). We treated a case for which SAP therapy was successful and prevented hypoglycemia. The patient was a baby boy who was small for his gestational age. He had hyperglycemia at 4 days of age, and a diagnosis of NDM had previously been made at another hospital. A continuous intravenous insulin infusion was initiated. At 29 days of age, the patient was transferred to our hospital for further treatment. SAP therapy was initiated at 39 days, which was successful and prevented hypoglycemia. Gradually, blood glucose levels improved. The insulin infusion was stopped to determine if any potential pump issues arose prior to discharge; the patient's blood glucose level did not increase. The decision was therefore made to discharge the patient from the hospital at 58 days of age with discontinued insulin. After discharge, genetic analysis showed hypomethylation on one of the alleles within 6q24, leading to a diagnosis of 6q24-related diabetes mellitus. Although almost all 6q24-related NDM cases are transient, no evidence exists for the appropriate timing of insulin discontinuation. Retrospective continuous glucose monitoring (CGM) analysis showed improved standard deviation (SD) values as well as improved blood glucose variability. This experience suggested SD values of CGM may be used as an index for tapering and discontinuing insulin in SAP therapy. However, future collaborative studies at other centers that focus on SD values as a guide for insulin discontinuation in SAP are required.

## 1. Introduction

Neonatal diabetes mellitus (NDM) is a rare genetic disease, occurring in one in 90,000 babies. Two types of NDM exist with distinct clinical courses: transient NDM (TNDM) and permanent NDM (PNDM) [1–3]. Insulin therapy is required for both TNDM and PNDM but can be withdrawn at between 3 and 18 months of age in most TNDM cases because of a gradual increase in endogenous insulin secretion. However, no prior studies have explored how to taper and terminate insulin doses. Sensor-augmented pump (SAP) therapy combines continuous subcutaneous insulin infusion (CSII) with continuous glucose monitoring (CGM). SAP therapy is thought to be useful in patients with type 1 diabetes and has been used in several patients with NDM in recent years [4, 5]. We report our experience of using SAP therapy in a patient with TNDM, where the standard deviation (SD) of the mean sensor glucose values in CGM was a useful indicator for when to taper and terminate insulin therapy.

## 2. Case Presentation

The patient was a 29-day-old baby boy with an unremarkable family history. He was diagnosed with fetal growth restriction and was born by induction at 40 weeks and 0 days. He was small for his gestational age, with a birth weight of 2,310 g (−2.8 SD), a height of 44.3 cm (−2.7 SD), and a head circumference of 32.5 cm (−0.69 SD). Blood glucose levels were monitored because of his low birth weight. Hyperglycemia of 413 mg/dL was recorded at 4 days of age, and he was subsequently transferred to another hospital. Blood analysis showed glucose at 377 mg/dL and insulin at 1.0 *μ*U/mL, leading to a diagnosis of NDM. A continuous intravenous insulin infusion was initiated, and his prefeeding blood glucose was maintained at between 100 and 200 mg/dL with 0.02 U/kg/h of insulin based on the patient's weight. C-peptide was 0.08 ng/mL at 15 days. At 29 days of age, he was transferred to our hospital for further treatment.

**Figure 1:**
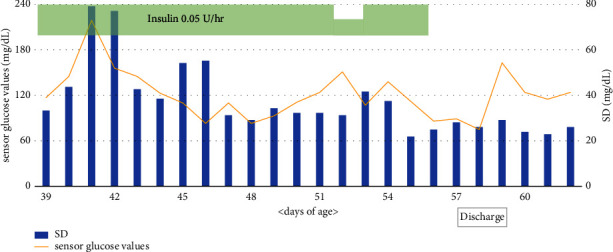
Sensor glucose values and standard deviation in CGM. CGM, continuous glucose monitoring; SD, standard deviation. Sensor glucose values indicated the average glucose values measured in CGM.

On admission, the patient presented with macroglossia and an umbilical hernia, and 6q24-related diabetes mellitus was suspected. SAP therapy was initiated at 39 days. A glucose sensor for CGM was attached to the abdomen, and a cannula for CSII was attached to the thigh. The patient weighed 3,355 g on the first day of SAP therapy, and therefore the insulin dose could be either 0.05 U/h or 0.075 U/h. We chose 0.075 U/h for the following reasons: (i) His blood glucose levels had been high, from 200 to 300 mg/dL, for several days; and (ii) the onset of insulin activity was expected to be slower when this was administered subcutaneously by insulin pump. The patient's blood glucose level dropped from 170 mg/dL to 70 mg/dL 2 h after initiating insulin at 0.075 U/h. We changed the dose to 0.05 U/h, which subsequently resulted in no hypoglycemia and an improvement in the patient's blood glucose control. A predictive low-glucose management (PLGM) system prevented the occurrence of hypoglycemia of less than 40 mg/dL during the patient's hospital stay. We educated the baby's parents on SAP therapy. We stopped insulin as a trial at 56 days to mimic the situation of a sudden interruption of insulin delivery after discharge that may cause catheter and other problems; however, the patient did not develop hyperglycemia. Therefore, CGM was continued, and the baby was discharged at 58 days of age. With no hyperglycemia thereafter, on a hospital visit at 63 days of age, the baby's CGM was discontinued (Figure 1). Genetic analysis showed hypomethylation within 6q24 on one of the alleles, leading to a diagnosis of 6q24-related diabetes mellitus.

## 3. Discussion

We found SAP therapy to be effective for our patient with TNDM for the following reasons: First, a PLGM system predicted hypoglycemia from CGM data and automatically suspended insulin delivery by CSII [5]. Few prior reports have described the effectiveness of a PLGM system in patients with TNDM [6]. Here, we demonstrated how a PLGM system could function correctly in an infant patient without causing hypoglycemia. Second, we hypothesized that assessing the SD of sensor glucose values in CGM may be useful for indicating when to taper and terminate insulin therapy. Retrospectively, the SD of sensor glucose values in CGM for our patient decreased after the experimental discontinuation of insulin therapy (Figure 1). A decrease in the SD may reflect the restoration of endogenous insulin secretion and, therefore, may be used to predict the timing of insulin withdrawal in patients with TNDM. A limitation is that the hypothesis is based on this case only. Further studies of more cases are necessary to determine insulin withdrawal criteria.

In conclusion, the concomitant use of a PLGM system enabled us to perform SAP therapy safely and to avoid hypoglycemia in a patient with TNDM. Our experience suggested that the SD of sensor glucose values in CGM was a useful indicator for when to taper and terminate insulin therapy.

## Data Availability

The data used to support the findings of this study are available from the author upon request.
